# Electrocardiographic Changes Associated with Early Repolarization Pattern in Healthy Young Males

**DOI:** 10.3390/medicina58081048

**Published:** 2022-08-04

**Authors:** Attila Frigy, Hunor Gábor-Kelemen, Szabolcs Attila László, István Adorján Szabó, Lóránd Kocsis

**Affiliations:** 1 Department of Cardiology, Clinical County Hospital Mures, 540103 Targu Mures, Romania; 2 Department of Internal Medicine IV, George Emil Palade University of Medicine, Pharmacy, Sciences and Technology of Targu Mures, 540103 Targu Mures, Romania; 3 Department of Pneumology, Clinical County Hospital Mures, 540103 Targu Mures, Romania

**Keywords:** early repolarization pattern, P wave, fragmented QRS, Tpeak-Tend interval

## Abstract

*Background and Objectives:* Early repolarization pattern (ERP) has recently been shown to be related with an increased risk of ventricular arrhythmias in susceptible individuals. *Materials and Methods:* We studied the ERP-associated ECG changes, with potential clinical relevance, in 220 young (age 22.1 ± 1.6 years), healthy, male subjects using 12-lead ECG recordings. A total of 38 subjects (17.3%) fulfilled the diagnostic criteria for ERP, and a total of 90 ECG characteristics were compared between the groups of subjects with and without ERP. *Results:* None of the ECGs were pathological, and 22 ECG parameters differed significantly (*p* < 0.05) between the subjects with and without ERP. Among them, the P wave-related parameters (e.g., average P wave duration: 101.5 ± 9.2 ms vs. 106.8 ± 9.9 ms, *p* = 0.004) and the presence of fragmented QRS complexes (67.6% vs. 92.1%, *p* = 0.002) revealed a potential propensity for atrial and ventricular arrhythmogenesis. The time-domain parameters of repolarization, those not corrected for QRS duration, showed shorter values (e.g., Tpeak-Tend interval: 70.9 ± 8.1 ms vs. 67.8 ± 8.0 ms, *p* = 0.036), reflecting the accelerated repolarization. *Conclusions:* Certain ECG characteristics seem to be more associated with ERP. The clinical significance of this finding at the individual level needs further prospective investigations.

## 1. Introduction

The early repolarization pattern (ERP) on the standard, 12-lead electrocardiogram (ECG) was recently recognized to be related with increased risk of ventricular arrhythmias and sudden cardiac death in susceptible individuals (early repolarization syndrome–ERS), mostly in the context of myocardial ischemia [1]. ERP is a form of J wave syndromes, which also include Brugada syndrome. Both conditions are caused by inherited ion-channell changes (chanellopaties) and are characterized by the appearance of prominent J waves on the surface ECG [2,3]. ERP could be found relatively frequently, in around 10% of the general population, with variable prevalence in various subpopulations, being more present in young males, athletes, physically active individuals, and African-American subjects [4,5].

**Figure 1:**
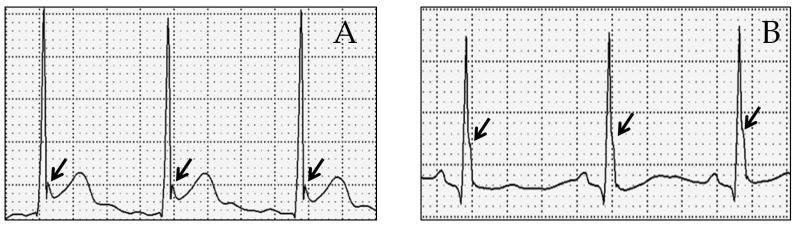
The appearance of J wave as notching at the end of the QRS complex (panel **A**, arrows), or slur on the descending limb of the positive R wave (panel **B**, arrows).

**Figure 2:**
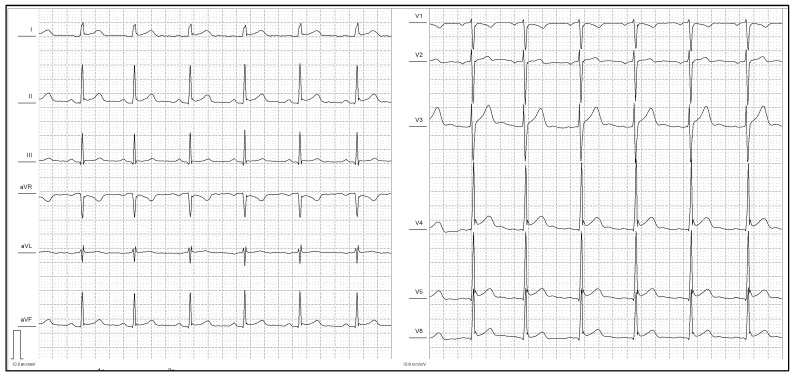
ECG with type 2 (notching at the end of QRS in the infero-lateral leads) ERP pattern and fQRS (I, aVL).

After decades of uncertainty, recent consensus papers and guidelines have established the diagnostic criteria for ERP: (1) presence of J wave-notching at the end of the QRS complex or slur on descending limb of the positive R wave-with or without ST-segment elevation—Figure 1; (2) J wave peak amplitude greater than 0.1 mV, present in ≥2 contiguous leads (inferior and/or lateral), except the V1-V3 leads; and (3) QRS duration less than 120 ms in the leads not containing notching or slur. Fulfilling all three criteria is required for diagnosis. ERP could be further classified as type 1 when the lateral leads are involved, type 2 when the inferior (±lateral) leads are involved, and type 3 when there is an involvement of all leads (including the right precordials) [6,7].

ERS is the very rare condition when ERP is associated with malignant ventricular arrhythmias and sudden cardiac death. On the basis of current evidence, ventricular arrhythmogenesis is related to intramural (phase 2) reentry caused by the enhanced transmural dispersion of repolarization. Risk stratification for ventricular arrhythmic events is a cornerstone issue in the presence of ERP. On the basis of ECG parameters, the risk of malignant ventricular arrhythmias is considered high in the case of: (1) ERP in the inferior or both in the inferior and lateral leads, (2) horizontal or descendent ST-elevation following the J wave, (3) increased J wave amplitude (≥2 mV), and (4) low T wave/R wave ratio [3,8,9,10,11].

Beyond the diagnosis and risk stratification of ERP, 12-lead ECG could reveal morphological and temporal characteristics—e.g., Brugada pattern, short or long QT interval, fragmented QRS, etc.—potentially related as markers or contributing factors to an enhanced arrhythmogenesis in these subjects. Because of the relative lack of data in the literature, we performed a systematic and complex examination of ERP-associated ECG changes to find out whether there are tendencies for more prevalent changes, with possible clinical relevance.

## 2. Materials and Methods

### 2.1. Study Population

The initial study population consisted of 241 healthy, young, male volunteers (mean age 22.1 ± 1.6 years) recruited from universities and high schools from the city of Targu Mures, Romania. There were no statistical differences between the groups in term of age, height, weight, body mass index, or body surface area (see Supplementary Table S3).

The subjects were free of any active or previous organ system (including cardiovascular) disease, having a negative clinical history, normal findings on clinical examination, not being on current medication, and showing normal sinus rhythm on the standard, 12-lead ECG. Due to technical reasons, 21 (8.7%) recordings were considered not suitable for analysis, 220 subjects being considered for final enrollment. The study was approved by the Ethical Committee of Research of the University of Medicine and Pharmacy of Targu Mures, Romania (CEC 129/2018), and all the subjects signed an informed consent regarding their participation.

### 2.2. Resting ECG Recordings: Diagnosis of ERP

In every subject, the resting, standard 12-lead ECG was recorded in a standardized manner: in supine position and basal conditions, after 10 min of resting, and avoiding smoking, caffeine containing drinks, alcohol, and eating at least 4 h before recording. The registrations were performed between 02:00 p.m. and 06:00 p.m. The hardware and software used for registering and processing the ECGs was a BTL-08 SD3 ECG device connected to a desktop PC, running the BTL CardioPoint ver. 2.27.24476.0 software. The sampling rate of analog–digital conversion was 2000 Hz, with a resolution of 13 bit. After excluding the tracings with recording artifacts, digital ECGs of 220 subjects were edited and analyzed. ERP was diagnosed on the standard 12-lead ECG based on the formerly presented recommendations from the 2015 and 2016 consensus documents [6,7].

### 2.3. ECG Parameters and Their Measurements

In every subject, a large number of morphological (waves, QRS axis, ST-T changes) and time–domain (heart rate, wave durations, segments, intervals) ECG parameters were measured and analyzed (90 items, totally). The complete list of parameters is presented in the Supplementary Table S1.

The measurements were made manually, in accordance with the current recommendations [12,13,14]. Heart rate-related intervals (RR, QT, Tpeak-Tend) were measured in every cycle and averaged. For measuring QT- and Tpeak-Tend-intervals, we considered the end of the T wave as the intersection of the isoelectric line and the tangent of the descendent portion of the T wave. The point of the maximal amplitude was considered the peak of the T-wave [15].

The presence of fragmented QRS was considered when in at least 2 contigous leads (with the exception of aVR), a QRS complex with duration <120 ms had notching of the R wave and/or the S wave (on the ascending or the descending part), not fulfilling the criteria for right or left bundle branch block. We also used the criteria of presence of a fragmented QRS complex in only one lead to have a better estimation of the phenomenon’s extension [16]. Bazett’s formula was used for correction with heart rate (xRR^1/2^) of the repolarization related intervals’ (QT, Tpeak-Tend) [13]. The manual measurements (intervals, amplitudes) were performed using the calipers of the ECG software, at the speed of 50 mm/s and at the amplification of 20 mm/mV of the tracing. The length of ECG recordings for analysis was 10 s. The analysis and measurement of the recordings and the quality control of the data were performed by the members of the research team, after a thorough learning process.

### 2.4. Statistical Analysis

Descriptive and comparative statistics were performed. Continuous variables were expressed as mean ± standard deviation, and the data distribution was tested by the Shapiro–Wilk test. The comparison between the groups of subjects with and without ERP was performed using Student’s *t*-test (for normal distribution) and Mann–Whitney U test (for non-normal distribution). Categorical variables were displayed as frequencies or percentages, and between-group comparisons were performed using the Chi-square test. A *p* value of <0.05 was considered statistically significant. Data processing was performed using Microsoft Excel (©Microsoft 2018), IBM SPSS Statistics 25 (©IBM Corporation 1994, 2018) and GraphPad Prism 7 (©GraphPad Software 1995–2017) software.

## 3. Results

Based on current diagnostic criteria, 17.3% (*n* = 38) of the subjects showed ERP on their ECG. Regarding the topographic distribution of the ERP, it was infero-lateral (type 2) in 30 cases, lateral (type 1) in 7 cases, and global (type 3) in only 1 case. Other details regarding the characteristics of ERP in the study population are presented in Table 1.

The J waves were found more frequently in the inferior leads. The slope of the ST-segment was predominantly upward in all leads, except the III lead, where the downward slope was dominant. The vast majority of the ERP-positive subjects fell into a low-risk category.

The statistically significant differences found by comparing the ECG parameters of subjects without (“ERP-” group, *n* = 182) and with (“ERP+” group, *n* = 38) ERP are presented in Table 2.

Definite pathological ECG changes were not found in the study population. The P wave-related parameters revealed an increased atrial depolarization duration and interatrial conduction time in the “ERP+” group. This was coupled with the more frequent appearance of atrial premature beats on the ECG recordings. The QRS duration was calculated till the onset of the J wave, hence it was shorter in the “ERP+” group, also causing the secondary increase of intervals normalized for QRS duration. The occurrence of fragmented QRS complexes (fQRS) was relatively frequent in the whole study population, being more prevalent in the “ERP+” group. Male type ST-segment elevation was found to be more common in the “ERP+” group. The existing significant differences between the parameters of repolarization reflected shorter intervals in the “ERP+” group. Certain ECG parameters differed in the two groups with borderline significance (intrinsicoid deflection, Sokolow–Lyon Index for right ventricular hypertrophy). The results of the comparisons are presented in more detail in Supplementary Table S2.

## 4. Discussion

The aim of our study was to find ECG changes associated with ERP as possible markers or contributors of enhanced arrhythmogenesis. We also considered that, up to now, a systematic, complex analysis of associated ECG changes in people with ERP is lacking in the literature. For the purpose of the study, we have enrolled young, healthy, male subjects, because of the higher prevalence of ERP in this population, assuming an inherent limitation of our work.

In subjects with ERP, the P wave-related results reflected a tendency of slower intra- and interatrial conduction, which is a well-known precursor of atrial arrhythmogenesis [17]. The incidental finding of more atrial ectopic beats in “ERP+” subjects is an interesting finding in this regard. The relationship of ERP and atrial fibrillation has been demonstrated in former studies, mutations of ion channel encoding genes, affecting both the atrial and ventricular electrophysiological properties, is responsible for this phenomenon [18,19]. On the other hand, regarding atrial morphofunctional changes, our former comparative echocardiographic study on healthy young male subjects with and without ERP did not demonstrate any difference, also underscoring the primacy of electrophysiological changes in atrial arrhythmogenesis in the subjects with ERP [20].

Male type ST-segment elevation (>1 mm in V2-4 leads, with upward concavity), a benign ECG sign, was found to be more frequent in subjects with ERP, a phenomenon which could be interpreted as part of accelerated repolarization.

Fragmented QRS complexes were found to be frequent in the study population, being more prevalent in the “ERP+” group (a representative example is shown on Figure 2). Generally, fQRS reflect local delays of ventricular depolarization and are related mainly to myocardial scar or fibrotic tissue [16].

fQRS were found to be significantly correlated with ventricular arrhythmogenesis in the setting of structural and electrical heart diseases (e.g., coronary artery disease, cardiomyopathies, Brugada syndrome) [21,22]. Moreover, the existing data confirm that fQRS are markers of enhanced arrhythmogenesis in patients with idiopathic ventricular fibrillation in the presence of J waves. [23,24]. It has to be emphasized that in healthy subjects, the presence of fQRS is not necessarily related to myocardial abnormalities, rather, it has the significance of local electrical conduction disturbances [25]. The demonstrated propensity of young male subjects with ERP for non-specific intraventricular conduction disturbances is likely to have a contributing role, in the case of their persistence, in future arrhythmic episodes in susceptible (e.g., genetically) individuals. Furthermore, this phenomenon underscores again the complexity of cardiac electrophysiological substrate in the setting of ERP. In our opinion, it is important, that the presence and topography of fQRS be noted when interpreting ECGs in subjects with ERP.

The parameters of repolarization did not differ significantly or reveal a shorter duration in the “ERP+” group. In ERP, gain-of-function mutations (KCNJ8, SCN1B β) and loss-of-function mutations (CACNA2D1, CACNA1C, CACNB2 and SCN5A) of ion channel encoding genes cause a net outward current during repolarization, which is responsible for the augmentation of the epicardial action potential notch [11]. These changes could explain the observed tendency for shorter duration of repolarization parameters. On the other hand, the Tpeak-Tend interval is largely recognized as a measure of transmural and spatial dispersion of repolarization [26]. The prolongation of the Tpeak-Tend interval has been found to be related to arrhythmogenesis both in Brugada syndrome and ERS [27,28]. Our finding, a shorter Tpeak-Tend interval in the group of subjects with ERP, underscores the failure of a mechanistic approach to repolarization analysis and suggests the important role of (shorter and longer term) dynamic factors in ERP-related ventricular arrhythmogenesis in susceptible individuals.

The selection criteria (age and gender) of the enrolled subjects represents a limitation of our study, however, for higher yield electrocardiograms regarding ERP, we have chosen a specific population. Evidently, the confirmation or disproof of our results in other populations (e.g., healthy women, healthy, elderly men, different patient categories, etc.) require further studies.

## 5. Conclusions

In healthy young men with ERP, compared to those without ERP, the existence of significant differences in certain ECG characteristics and parameters was demonstrated, revealing a potential predisposition to atrial and ventricular arrhythmogenesis. However, determining the clinical significance of the observed changes at the individual level needs further investigations, potentially including machine learning in the analysis of data from large populations. One of the principal aims of our study was to draw attention to the importance of comprehensive ECG analysis in the setting of ERP. Searching for markers of cardiac arrhythmia propensity is always important, regardless of demographic characteristics or clinical situation.

## Figures and Tables

**Table 1:** The main characteristics of the ERP pattern in the study population.

**The Dominant Appearance of J Wave**	**Slurring**	**Notching**	
Dominant appearance (in ≥2 contiguous leads) of J wave in the lateral leads (n)	4	12	
Dominant appearance (in ≥2 contiguous leads) of J wave in the inferior leads (n)	13	18	
**The type of ST segment slope in leads presenting ERP**	**Horizontal**	**Downward**	**Upward**
ST segment slope in lateral leads (n)	7	3	36
ST segment slope in inferior leads (n)	13	25	54

Lateral leads (I, aVL, V5,6) are involved in type 1, type 2, and type 3 ERP; inferior leads (II, III, aVF) are involved in type 2 and type 3 ERP. The ST segment slope was defined in all leads where the J wave was >0.1 mV. All data are displayed as frequencies.

**Table 2:** The significant differences found by comparing ECG parameters of subjects without (“ERP-”) and with (“ERP+”) ERP.

ECG Parameter	“ERP-” Group (*n* = 182)	“ERP+” Group (*n* = 38)	Difference between Groups	*p*-Values
Negative terminal P in inferior lead (s) (%)	19.3	34.2	+14.9	0.044
Longest P wave in I, II, III, aVL, aVF (PDurmax) (ms)	110.4 ± 10.2	114.2 ± 9.4	+3.8	0.032
Average P wave duration (ms)	101.5 ± 9.2	106.8 ± 9.9	+5.3	0.004
Fragmented QRS (notching of QRS <120 ms in at least one lead, excepting aVR) (%)	67.6	92.1	+24.5	0.002
Fragmented QRS (notching of QRS <120 ms in at least two contigous leads, excepting aVR) (%)	49.5	73.7	+24.2	0.006
Intrinsicoid deflection in V5 (ms)	43.4 ± 7.3	41.0 ± 6.3	−2.4	0.045
QRS duration in II (ms)	92.2 ± 15.3	76.9 ± 11.9	−15.33	<0.001
QRS duration in V2 (ms)	96.8 ± 11.9	91.8 ± 8.6	−5.0	0.003
QRS duration in V5 (ms)	92.1 ± 12.9	78.5 ± 9.4	−13.6	<0.001
Max of QRS duration from lead II, V2, V5 (ms)	98.7 ± 11.8	92.5 ± 8.0	−6.2	<0.001
Sokolow-Lyon index for RVH (R in V1 or V2 + S in V5 or V6) (mm)	11.0 ± 4.0	9.7 ± 3.3	−1.3	0.043
Male type ECG pattern (ST-elevation > 1 mm in V2-4) (%)	38.6	57.9	+19.3	0.029
QT max/QRS in V5	4.1 ± 0.6	4.7 ± 0.6	+0.6	<0.001
QT max corrected/QRS in V5	4.5 ± 0.8	5.4 ± 1.1	+0.9	<0.001
QT dispersion = difference of max and min QT in I, aVF, V2 (ms)	25.1 ± 12.5	16.7 ± 9.9	−8.4	<0.001
QT dispersion corrected (Bazett’s formula) (ms)	26.2 ± 12.5	17.9 ± 11.4	8.3	<0.001
QT dispersion/QT average	0.070 ± 0.034	0.046 ± 0.028	0.024	<0.001
Tpeak—Tend average (ms)	70.9 ± 8.1	67.8 ± 8.0	3.1	0.036
Tpeak—Tend average corrected (Bazett’s formula) (ms)	77.1 ± 10.4	72.4 ± 10.5	4.7	0.016
Tpeak—Tend max corrected (Bazett’s formula) (ms)	90.3 ± 14.6	83.4 ± 13.1	−6.9	0.005
Tpeak—Tend average/QT average	0.202 ± 0.022	0.190 ± 0.019	−0.012	0.001
Atrial premature beats (%)	0.6	7.9	+7.3	0.011

Data are presented as mean ± standard deviation (continuous variables) or as percentages (categorical variables). Abbreviations: RVH—right ventricular hypertrophy.

## Data Availability

The dataset used for the current study is available from the corresponding author on reasonable request.

## Supplementary Materials

The following are available online at https://www.mdpi.com/article/10.3390/medicina58081048/s1, Table S1: The list of analyzed ECG-parameters, Table S2: The results of the compared ECG parameters of subjects without (“ERP-”) and with (“ERP+”) ERP, Table S3: The basic characteristics of subjects without (“ERP-”) and with (“ERP+”) ERP.
