# Supplementary material for: Electrocardiographic Changes Associated with Early Repolarization Pattern in Healthy Young Males
Source: Medicina (Kaunas). 2022 Aug 4;58(8):1048. doi: 10.3390/medicina58081048 (PMC9415057; doi:10.3390/medicina58081048)
Supplement: Supplementary file 1 [file medicina-58-01048-s001.zip › Supplementary Table S1.pdf]

Supplementary Table S1 The list of analyzed ECG-parameters. The ECG measurements were performed at amplification of 20 mm/mV, and speed of 50 mm/s

**ECG parameter**	**Measurement units or categories**	
**Heart rate module**		
Mean RR interval		ms
Mean heart rate		beats/min
**QRS axis module**		
Position		grades
Intermediate (30-60 gr)	Yes	No
Horizontalized (-30-30 gr)		
Verticalized (60-90 gr)		
Extreme left, left anterior hemibloc		
Extreme right, left posterior hemibloc		
No man`s land (-90-180 gr)		
**P wave module**		
Sinusal P wave	Yes	No
Retrograde P in inferior leads		
Wandering pacemaker (≥ 3 P wave morphology)		
Bifid P wave in limb leads		
Negative terminal P in inferior lead(s) (Bayes` sign – interatrial block)		
Biphasic P wave in V1		
**P wave amplitude module**		
Highest P wave amplitude in I, II, III, aVL, aVF (PAmax)		mm
Smallest P wave amplitude in I, II, III, aVL, aVF (PAmin)		
PAmax-PAmin (P wave amplitude dispersion)		
Amplitude of positive component of P wave in V1		
Amplitude of negative component of P wave in V1		
**P wave duration (dur) module**		
Longest P wave in I, II, III, aVL, aVF (Pdurmax)		ms
Shortest P wave in I, II, III, aVL, aVF (Pdurmin)		ms
P wave duration dispersion (Pdurmax-Pdurmin)		ms
Average P wave duration in limb leads		ms
PQ (PR) interval (the longer in I, II, III, aVL, aVF)		ms
**QRS module**		
Q wave in lateral (I, aVL, V5,6) leads	Yes	No
Q wave in inferior (II, III, aVF) leads		
Q wave amplitude in any lead > 2 mm		
Complete LBBB pattern		
Incomplete LBBB pattern (Q wave absent in V5,6)		

Complete RBBB pattern		
Incomplete RBBB pattern		
Non-specific intraventricular conduction disturbance (fragmentedQRS)		
Late R/S transition (R<S in V4) in precordial leads		
Type A ventricular preexcitation (predominant R in V1)		
Type B ventricular preexcitation (predominant R in V5)		
**QRS duration module**		
Intrinsicoid deflection in aVL		ms
Intrinsicoid deflection in V5		
QRS duration in II		
QRS duration in V2		
QRS duration in V5		
**QRS amplitude module**		
Sokolow-Lyon index (S in V1 or V2 + R in V5 or V6)		mm
Sokolow-Lyon index (R in V1 or V2 + S in V5 or V6)		
Cornell index (R in aVL+ S in V3)		
max amplitude of R wave in V4, V5, V6		
**ERP module**		
Maximal amplitude of J-point elevation		mm
ERP (slur or notching) in II, III, aVF	Yes	No
ERP (slur or notching) in V4-6		
ERP (slur or notching) in II, III, aVF, V4-6		
Slur (and leads with slur)		
Notching (and leads with notching)		
Male type ECG (ST-elevation > 1 mm in V2-4)		
ST elevation > 1 mm in other leads		
Type I Brugada pattern (including + 1 intercostal space superiorly)		
Type II Brugada pattern (including + 1 intercostal space superiorly)		
Type III Brugada pattern (including + 1 intercostal space superiorly)		
**T-wave module**		
Juvenile ECG pattern (negative T in V1, 2)	Yes	No
Tall T waves (>2/3 R in positive QRS leads) in lateral leads		
Tall T waves (>2/3 R in positive QRS leads) in inferior leads		
Tall T waves in negative QRS leads		
Flat T waves (<± 1mm) in lateral leads		
Flat T waves (<± 1mm) in inferior leads		
Flat T waves (<± 1mm) in anterior leads		
Diffuse flat T waves (<± 1mm)		
Negative T waves (> - 1mm) in lateral leads		

Negative T waves (> - 1mm) in inferior leads		
Negative T waves (> - 1mm) in anterior leads		
**ST segment module**		
ST depression (> 0,5 mm) in lateral leads	Yes	No
ST depression (> 0,5 mm) in inferior leads		
ST depression (> 0,5 mm) in anterior leads		
Diffuse ST depression (>0.5 mm)		
Epsilon wave in V1		
**QT measurements (II, V2, V5, without leads with T wave amplitude <1,5 mm) module**		
QT average		ms
QT average corrected (Bazett`s formula)		
QT max		
QT max corrected (Bazett`s formula)		
QTmax/QRS in V5		
Qtmax corrected/QRS in V5		
QT dispersion = difference of max and min QT in leads *I, aVF, V2*		
QT dispersion corrected (Bazett`s formula)		
QT dispersion/QT average		
**Tpeak-Tend measurements (II, V2, V5, without leads with T wave amplitude <1,5 mm) module**		
Tpeak-Tend average		ms
Tpeak-Tend average corrected (Bazett`s formula)		
Tpeak-Tend max		
Tpeak-Tend max corrected (Bazett`s formula)		
Tpeak-Tend average/ QT average		
Tpeak-Tend average corrected/ QT average corrected		
**Arrhythmia module**		
Atrial premature beats(s)	Yes	No
Ventricular premature beat(s) of RV orign (LBBB pattern)		
Ventricular premature beat(s) of LV orign (RBBB pattern)		

Abbreviations: LBBB – left boundle branch block, RBBB – right boundle branch block, RVH – right ventricular hypertrophy, LVH – left ventricular hypertrophy, ERP – early repolarization pattern
