# Supplementary material for: Electrocardiographic Changes Associated with Early Repolarization Pattern in Healthy Young Males
Source: Medicina (Kaunas). 2022 Aug 4;58(8):1048. doi: 10.3390/medicina58081048 (PMC9415057; doi:10.3390/medicina58081048)
Supplement: Supplementary file 1 [file medicina-58-01048-s001.zip › Supplementary Table S2.pdf]

Supplementary Table S2. The results of the compared ECG parameters of subjects without (“ERP-”) and with (“ERP+”) ERP. The statistically significant differences are marked with bold.

ECG Parameter	unit	"ERP-" Group (n = 182)	"ERP+" Group (n = 38)	Difference between groups	p-Value
		**Heart rate module**			
Mean Heart rate	beats/min	71.9 ± 11.7	68.9 ± 10.3	-3.0	0.112
Mean RR interval	ms	857.4 ± 145.0	891.7 ± 144.2	-34.3	0.190
		**QRS axis module**			
QRS axis	grade	62.6 ± 16.8	64.9 ± 17.3	+2.3	0.488
Intermediate (30-60 grades)	%	27.3 %	28.9 %	+1.6 %	0.834
Horizontalized (-30-30 grades)	%	11.4 %	5.3 %	-6.1 %	0.227
Verticalized (60-90 grades)	%	54.5 %	57.8 %	+3.3 %	0.707
Extreme left, left anterior hemiblock	%	1.7 %	2.6 %	+0.9 %	0.715
Extreme right, left posterior hemiblock	%	4.0 %	5.3 %	+1.3 %	0.728
No man`s land (-90-180 gardes)	%	0.6 %	0.0 %	-0.6 %	0.531
		**P wave module**			
Sinusal P	%	98.9 %	100.0 %	+1.1 %	0.375
Retrograde P in inferior leads	%	0.6 %	5.3 %	+4.7 %	0.059
Wandering pacemaker (≥ 3 P wave morphology)	%	3.4 %	5.3 %	+1.9 %	0.601
Bifid P wave in limb leads	%	40.6 %	45.9 %	+5.3 %	0.547
**Negative terminal P in inferior lead(s)**	**%**	**19.3 %**	**34.2 %**	**+14.9 %**	**0.044**
Biphasic P wave in V1	%	64.2 %	63.2 %	-1.0 %	0.903
Highest P wave amplitude in I, II, III, aVL, aVF (PA max)	mm	1.3 ± 0.4	1.2 ± 0.4	-0.1	0.176
Smallest P wave amplitude in I, II, III, aVL, aVF (PA min)	mm	0.350 ± 0.096	0.352 ± 0.090	+0.002	0.932
Pamplitude max - min	mm	0.936 ± 0.401	0.858 ± 0.394	-0.078	0.273
**Longest P wave I, II, III, aVL, aVF (PDur max)**	**ms**	**110.4 ± 10.2**	**114.2 ± 9.4**	**+3.8**	**0.032**
Shortest P wave I, II, III, aVL, aVF (PDur min)	ms	86.4 ± 14.3	88.8 ± 14.9	+2.4	0.384
PDur max - PDur min	ms	24.2 ± 13.8	24.8 ± 16.1	+0.6	0.848
**Average P wave duration**	**ms**	**101.5 ± 9.2**	**106.8 ± 9.9**	**+5.3**	**0.004**
Amplitude of positive component of P wave in V1	mm	0.456 ± 0.198	0.424 ± 0.205	-0.031	0.407

Amplitude of negative component of P wave in V1	mm	0.308 ± 0.202	0.288 ± 0.194	-0.020	0.644
PQ (PR) interval (the longeST in I, II, III, aVL, aVF)	ms	160.4 ± 19.8	159.1 ± 16.1	-1.3	0.654
**QRS module**					
Q wave in lateral (I, aVL, V5,6) leads	%	82.4 %	84.2 %	+1.8 %	0.787
Q wave in inferior (II, III, aVF) leads	%	77.3 %	84.2 %	+6.9 %	0.345
Q wave amplitude > 2 mm	%	13.1 %	13.2 %	+0.1 %	0.998
Major LBBB pattern	%	0.0 %	0.0 %	0.0 %	-
Minor LBBB pattern	%	2.8 %	0.0 %	-2.8 %	0.159
Major RBBB pattern	%	3.4 %	0.0 %	-3.4 %	0.123
Minor RBBB pattern	%	10.9 %	4.6 %	-6.3 %	0.221
**Fragmented QRS (notching of QRS <120 ms in at least one lead, excepting aVR)**	%	**67.6 %**	**92.1 %**	**+24.5 %**	**0.002**
Late R/S transition (R<S in V4) in precordial leads	%	6.3 %	10.5 %	+4.2 %	0.373
Type A ventricular preexcitation (dominant R in V1)	%	0.0 %	0.0 %	0.0 %	-
Type B ventricular preexcitation (dominant R in V5)	%	0.6 %	0.0 %	-0.6 %	0.531
Intrinsecoidal deflection in aVL	ms	36.0 ± 10.7	34.5 ± 12.7	-1.5	0.491
**Intrinsicoidal deflection in V5**	**ms**	**43.4 ± 7.3**	**41.0 ± 6.3**	**-2.4**	**0.045**
**QRS duration in II**	**ms**	**92.2 ± 15.3**	**76.9 ± 11.9**	**-15.3**	**<0.001**
**QRS duration in V2**	**ms**	**96.8 ± 11.9**	**91.8 ± 8.6**	**-5.0**	**0.003**
**QRS duration in V5**	**ms**	**92.1 ± 12.9**	**78.5 ± 9.4**	**-13.6**	**<0.001**
**Max QRS duration from lead II, V2, V5**	**ms**	**98.7 ± 11.8**	**92.5 ± 8.0**	**-6.2**	**<0.001**
Sokolow-Lyon index for LVH (S in V1 or V2 + R in V5 or V6)	mm	34.6 ± 9.1	36.8 ± 6.0	+2.2	0.063
**Sokolow-Lyon index for RVH (R in V1 or V2 + S in V5 or V6)**	**mm**	**11.0 ± 4.0**	**9.7 ± 3.3**	**-1.3**	**0.043**
Cornell index for LVH (R in aVL+ S in V3)	mm	14.1 ± 5.8	12.7 ± 5.4	-1.4	0.150
Max R wave amplitude in V4, V5, V6	mm	19.7 ± 5.2	19.2 ± 5.8	-0.5	0.629
**ST-T, QT, Tpeak-Tend module**					
Juvenile ECG pattern (negative T in V1,2)	%	45.5 %	42.1 %	-3.4 %	0.707
**Male type ECG (ST-elevation > 1 mm in V2-4)**	%	**38.6 %**	**57.9 %**	**+19.3 %**	**0.029**
Tall T waves (>2/3 R in positive QRS leads) in lateral leads	%	0.6 %	0.0 %	-0.6 %	0.535

Tall T waves (>2/3 R in positive QRS leads) in inferior leads	%	0.0 %	0.0 %	0.0 %	-
Tall T waves in negative QRS leads	%	0.0 %	0.0 %	0.0 %	-
ST depression (> 0,5 mm) in lateral leads	%	0.0 %	0.0 %	0.0 %	-
ST depression (> 0,5 mm) in inferior leads	%	3.4 %	0.0 %	-3.4 %	0.123
ST depression (> 0,5 mm) in anterior leads	%	0.0 %	0.0 %	0.0 %	-
Diffuse ST depression (>0.5 mm)	%	0.0 %	0.0 %	0.0 %	-
Flat T waves (< ± 1mm) in lateral leads	%	1.7 %	7.9 %	+6.2 %	0.067
Flat T waves (< ± 1mm) in inferior leads	%	7.4 %	7.9 %	+0.5 %	0.915
Flat T waves (<± 1mm) in anterior leads	%	0.0 %	2.6 %	+2.6 %	0.062
Diffuse flat T waves (< ± 1mm)	%	0.0 %	0.0 %	0.0 %	-
Negative T waves (> - 1mm) in lateral leads	%	0.6 %	2.6 %	+2.0 %	0.297
Negative T waves (> - 1mm) in inferior leads	%	0.6 %	2.6 %	+2.0 %	0.297
Negative T waves (> - 1mm) in anterior leads	%	0.6 %	0.0 %	-0.6 %	0.531
Epsilon wave in V1	%	1.1 %	0.0 %	-1.1 %	0.375
QT average	ms	353.0	356.8	+3.8	0.365
QT average corrected (Bazett`s formula)	ms	383.3	380.3	-3.0	0.497
QT max	ms	365.9	369.5	+3.6	0.388
QT max corrected (Bazett`s formula)	ms	397.7	393.9	-3.8	0.476
**QT max/QRS in V5**		**4.1 ± 0.6**	**4.7 ± 0.6**	**+0.6**	**<0.001**
**Qt max corrected/QRS in V5**		**4.5 ± 0.8**	**5.4 ± 1.1**	**+0.9**	**<0.001**
**QT dispersion = difference of max and min QT in *I, aVF, V2***	**ms**	**25.1 ± 12.5**	**16.7 ± 9.9**	**-8.4**	**<0.001**
**QT dispersion corrected (Bazett`s formula)**	**ms**	**26.2 ± 12.5**	**17.9 ± 11.4**	**-8.3**	**<0.001**
**QT dispersion/QT average**		**0.070 ± 0.034**	**0.046 ± 0.028**	**-0.024**	**<0.001**
**Tpeak – Tend average**	**ms**	**70.9 ± 8.1**	**67.8 ± 8.0**	**-3.1**	**0.036**
**T peak– Tend average corrected (Bazett`s formula)**	**ms**	**77.1 ± 10.4**	**72.4 ± 10.5**	**-4.7**	**0.016**
Tpeak – Tend max	ms	81.5 ± 9.4	78.6 ± 9.7	-2.9	0.101
**Tpeak -Tend max corrected (Bazett`s formula)**	**ms**	**90.3 ± 14.6**	**83.4 ± 13.1**	**-6.9**	**0.005**
**Tpeak – Tend average/ QT average**		**0.202 ± 0.022**	**0.190 ± 0.019**	**-0.012**	**0.001**
**Arrhythmia module**					
**Atrial premature beats**	%	**0.6 %**	**7.9 %**	**+7.3 %**	**0.011**
Ventricular premature beatS of RV orign (LBBB pattern)	%	0.0 %	2.6 %	+2.6 %	0.062

Ventricular premature beats of LV orign (RBBB pattern)	%	1.7 %	0.0 %	-1.7 %	0.418

Abbreviations: LBBB – left boundle branch block, RBBB – right boundle branch block, RVH – right ventricular hypertrophy, LVH – left ventricular hypertrophy
