# Supplementary material for: Electrocardiographic Changes Associated with Early Repolarization Pattern in Healthy Young Males
Source: Medicina (Kaunas). 2022 Aug 4;58(8):1048. doi: 10.3390/medicina58081048 (PMC9415057; doi:10.3390/medicina58081048)
Supplement: Supplementary file 1 [file medicina-58-01048-s001.zip › Supplementary Table S3.pdf]

Supplementary Table S3. The basic characteristic of subjects without (“ERP-”) and with (“ERP+”) ERP.

ECG Parameter	unit	"ERP-" Group (n = 182)	"ERP+" Group (n = 38)	Difference between groups	p-Value
Age	year	21.9 ± 1.7	22.2 ± 1.5	0.3	0.376
Height	cm	179.7 ± 7.2	180.0 ± 7.6	0.3	0.819
Weight	kg	75.5 ± 11.4	77.0 ± 10.3	1.5	0.518
Body mass index	kg/m^2	23.2 ± 2.7	23.7 ± 3.0	0.5	0.355
Body surface area	m^2	1.93 ± 0.16	1.95 ± 0.14	0.02	0.434
